# Ticagrelor vs. Clopidogrel After Complex Percutaneous Coronary Intervention in Patients With Stable Coronary Artery Disease

**DOI:** 10.3389/fcvm.2021.768190

**Published:** 2021-11-22

**Authors:** Ziwei Xi, Jianan Li, Hong Qiu, Tingting Guo, Yong Wang, Yang Li, Jianfeng Zheng, Kefei Dou, Bo Xu, Yongjian Wu, Shubin Qiao, Weixian Yang, Yuejin Yang, Runlin Gao

**Affiliations:** ^1^Department of Cardiology, Coronary Artery Disease Center, State Key Laboratory of Cardiovascular Disease, Fuwai Hospital, National Center for Cardiovascular Diseases, Peking Union Medical College, Chinese Academy of Medical Sciences, Beijing, China; ^2^Department of Cardiology and Macrovascular Disease, Beijing Tiantan Hospital, Capital Medical University, Beijing, China; ^3^State Key Laboratory of Cardiovascular Disease, Fuwai Hospital, National Center for Cardiovascular Diseases, Peking Union Medical College, Chinese Academy of Medical Sciences, Beijing, China; ^4^Thrombosis Center, State Key Laboratory of Cardiovascular Disease, Fuwai Hospital, National Center for Cardiovascular Diseases, Peking Union Medical College, Chinese Academy of Medical Sciences, Beijing, China

**Keywords:** ticagrelor, clopidogrel, antiplatelet therapy, complex PCI, stable coronary artery disease

## Abstract

**Background:** Patients undergoing complex percutaneous coronary intervention (PCI) have an increased risk of cardiovascular events. Whether potent antiplatelet therapy after complex PCI improves outcomes in patients with stable coronary artery disease (SCAD) remains unclear.

**Objectives:** To assess the efficacy and safety of ticagrelor *vs*. clopidogrel in patients with SCAD undergoing complex PCI.

**Methods:** Patients with a diagnosis of SCAD and undergoing PCI during January 2016 to December 2018 were selected from an institutional registry. The primary efficacy endpoint was major adverse cardiac events (MACE) within 12 months after PCI. The primary safety endpoint was major bleeding.

**Results:** Among 15,459 patients with SCAD included in this analysis, complex PCI was performed in 6,335 (41.0%) patients. Of patients undergoing complex PCI, 1,123 patients (17.7%) were treated with ticagrelor. The primary efficacy outcome after complex PCI occurred in 8.6% of patients in the ticagrelor group and 11.2% in the clopidogrel group. Compared with clopidogrel, ticagrelor decreased the risk of MACE in patients undergoing complex PCI [adjusted hazard ratio (HR): 0.764; 95% confidence interval (CI): 0.615 to 0.949; *p* = 0.015], but not in non-complex PCI (*p* for interaction = 0.001). There was no significant difference in incidence of major bleeding between patients treated with ticagrelor and clopidogrel (*p* = 0.221), while ticagrelor was associated with an increased risk of minor bleeding (adjusted HR: 3.099; 95% CI: 2.049 to 4.687; *p* < 0.001).

**Conclusion:** In patients with SCAD and undergoing complex PCI, ticagrelor could substantially reduce the risk of adverse cardiovascular outcomes without increasing the risk of major bleeding compared with clopidogrel.

## Introduction

Dual antiplatelet therapy, consisting of aspirin and a P2Y12 receptor inhibitor, is the standard of care for patients undergoing percutaneous coronary intervention (PCI) to prevent atherothrombotic events (1). Ticagrelor is an oral, direct-acting, reversible platelet inhibitor (*via* P2Y12 receptor), which can provide more potent inhibition of platelets than clopidogrel, a broadly utilized traditional P2Y12 receptor inhibitor (2). In patients treated with stent implantation, current guidelines favor potent platelet inhibition with ticagrelor over clopidogrel in patients with acute coronary artery syndrome because of its superior net clinical benefits, while clopidogrel is indicated for the treatment of patients with stable coronary artery disease (SCAD) (1, 3). However, there is a lack of studies investigating the efficacy and safety of ticagrelor *vs*. clopidogrel in patients with SCAD.

Practice guidelines suggested that ticagrelor should be considered in selected patients with SCAD with higher risk of ischemic events (1, 3). It is noteworthy that the greater benefits of the more potent P2Y12 inhibitors ticagrelor in previous studies come at the cost of a higher risk of bleeding compared with clopidogrel (4, 5). Therefore, it is important to balance between the risk of ischemia and bleeding and identify patients who might benefit from potent antiplatelet therapy in order to improve the outcomes of patients with SCAD after PCI.

Currently, increasing numbers of PCIs are performed in patients with complex anatomic features (6). The increment of procedural complexity is associated with a higher risk of ischemic events in patients undergoing PCI. The procedural complexity has been proposed to be an important parameter to take into consideration when tailoring DAPT (7). Whether more intense antithrombotic therapies after complex PCI could provide stronger protection against adverse cardiac events without increasing bleeding risk in patients with SCAD is unclear. Hence, the present study aimed to determine the optimal choice of P2Y12 inhibitor in patients with SCAD and undergoing complex PCI by assessing the efficacy and safety of ticagrelor compared with clopidogrel.

## Methods

### Study Design and Population

The present study is a retrospective cohort analysis of data from the efficacy and safety of genetic and platelet function testing for guiding antiplatelet therapy after percutaneous coronary intervention (GF-APT) registry (ChiCTR2100047090). The GF-APT is a single-center registry, which retrospectively enrolled a total of 41,090 consecutive patients treated with PCI successfully during the index hospitalization and discharged with DAPT in Fu Wai Hospital between January 2016 and December 2018. In the GF-APT, data on de-identified patient demographics, medical history, results of laboratory assessments, angiographic features, procedural characteristics, and information of treatment were collected retrospectively from electronic medical records for all enrolled patients. There was no treatment intervention directed by the protocol in the registry. All patients enrolled in the GF-APT were followed up for at least 1 year.

Among patients participating in this registry, patients aged 18 years or older and presenting with SCAD at admission were identified and selected to constitute the present study population. The patients were eligible if they had stable angina and evidence of coronary artery disease (CAD) defined by at least one of the following criteria: (1) prior myocardial infarction (>12 months ago), (2) prior coronary revascularization (> 12 months ago), or (3) stenosis of ≥ 50% of the luminal diameter of at least one native vessel detected by coronary angiography (8). The major exclusion criteria of our study were the following: (1) indications of long-term treatment with oral anticoagulants, (2) any contraindication to aspirin or P2Y12 receptor inhibitors, including ticagrelor and clopidogrel, (3) life expectancy of <1 year, (4) expected duration of DAPT <6 months.

Included patients were divided into complex the PCI group and the non-complex PCI group according to procedural complexity. Furthermore, patients in complex and non-complex PCI groups were respectively divided into the clopidogrel group and the ticagrelor group in accordance with choice of the P2Y12 receptor inhibitor administered after PCI. Treatment with either clopidogrel or ticagrelor was identified at discharge or at the time of that in-hospital event if an adverse event occurred before discharge. The definition of complex PCI in the present study has been utilized in previously published studies (7, 9). Briefly, PCI would be identified as complex PCI when it met any one of the following characteristics: three vessels treated, ≥three stents implanted, ≥three lesions treated, bifurcation PCI with ≥two stents, total stent length > 60 mm, unprotected left main PCI, or surgical bypass graft or chronic total occlusion as target lesions.

This study was approved by the ethics committee of Fu Wai Hospital, complying with the principles of the Declaration of Helsinki, and written informed consent was waived because the patient data were de-identified, and this was a non-interventional study.

### Follow-Up and Study Endpoints

All the patients were followed up to 12 months or until the time of an event. Follow-up was performed by telephone interviewers using standardized questionnaires at 6 and 12 months after the index procedure. The primary efficacy endpoint of the study was major adverse cardiac events (MACE), defined as a composite of cardiac death, myocardial infarction (MI), and repeat target vessel revascularization. Cardiac death included any death caused by cardiac disease or the cause of which could not be determined by a physician. The definition of MI was consistent with the Third Universal Definition of Myocardial Infarction. (10) The primary safety endpoint was a major bleeding event, defined as Bleeding Academic Research Consortium criteria type 3 or type 5 bleeding event (11). The secondary endpoints were cardiac death, MI, repeat target vessel revascularization, and minor bleeding events. Minor bleeding was defined as a bleeding event that required non-surgical medical intervention by a healthcare professional, led to an increased level of care, or prompted evaluation.

### Statistical Analysis

Baseline characteristics of patients treated with ticagrelor were compared with patients treated with clopidogrel and stratified for complex PCI. Categorical variables were reported counts (proportions) and were compared using the chi-square test or Fisher's exact test as appropriate. Continuous variables were summarized as mean ± standard deviation or median [interquartile range (IQR)] and compared using the independent sample *t*-test or Mann–Whitney test based on their distributions.

The cumulative incidence of clinical outcomes up to 1 year was evaluated by the Kaplan–Meier method and compared using the log-rank test. Cox proportional hazard models were used to calculate hazard ratio (HR) and 95% confidence intervals (CI) to compare the efficacy and safety outcomes of ticagrelor *vs*. clopidogrel. To adjust for non-randomized selection of treatment, the following covariates, which have been reported to be risk factors in adverse outcomes after PCI by previous literature, were included in the multivariate analyses: age, sex, body mass index, hypertension, hyperlipidemia, diabetes, ever-smoking, prior MI, prior PCI, prior ischemic stroke, and renal insufficiency. Additionally, covariates that differed between the two groups with statistical significance (*p* < 0.1) in the univariable analysis were also used to construct the multivariate models. We assessed the statistical significance of possible heterogeneity in the treatment effect of ticagrelor and clopidogrel between complex and non-complex PCI subgroups by adding an interaction term to the multivariate Cox regression models. An additional sensitivity analysis was performed by excluding patients who switched between ticagrelor and clopidogrel during follow-up before assessing the association between ticagrelor or clopidogrel and clinical outcomes. The impact of each component of complex PCI on the adverse outcome was also estimated with multivariate Cox regression models.

All reported *p* values are two-sided, and *p* values < 0.05 were considered as statistically significant for all analyses. Statistical analyses were performed using IBM SPSS Statistics version 25 (SPSS, Chicago, IL).

## Results

**Figure 1:**
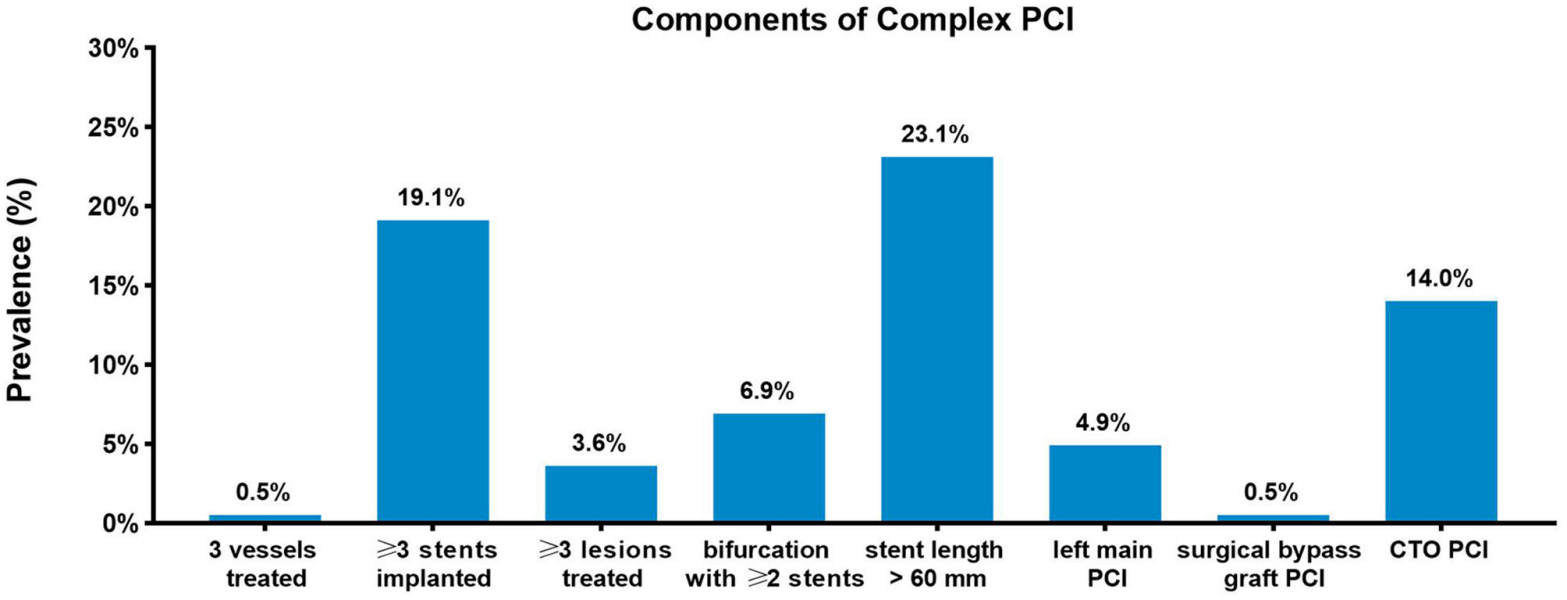
Prevalence of complex percutaneous coronary intervention components. Prevalence of each complex percutaneous coronary intervention (PCI) component in the study population.

The study population consisted of 15,459 patients diagnosed as stable CAD from the registry. Among these included patients, 6,335 (41.0%) underwent complex PCI, whereas 9,124 underwent non-complex PCI. Baseline characteristics according to procedural complexity are presented in Table 1, and significant differences were observed between complex and non-complex PCI groups. Patients who underwent complex PCI were more likely to be male and had higher BMI. The proportions of smokers and patients who had diabetes mellitus were higher in the complex PCI group. The patients with complex PCI more commonly have suffered previous MI and had a history of revascularization. With respect to medication at discharge, statin, beta-blocker, and ACEI/ARB were more frequently prescribed to patients undergoing complex PCI. The prevalence of each complex PCI component in the overall study population is presented in Figure 1. Of all included patients, 99.7% were treated with drug-eluting stents, 0.2% (*n* = 35) with new-generation bioresorbable stents, and 0.1% (*n* = 11), with bare metal stents.

**Table 1:** Baseline characteristics of patients undergoing complex vs. non-complex PCI.

	**Complex PCI (*n* = 6,335)**	**Non-complex PCI (*n* = 9,124)**	***p* Value**
Age, years	59.1 ± 10.1	59.3 ± 9.9	0.258
Female	1,249 (19.7%)	2,214 (24.3%)	<0.001
Body mass index, kg/m^2^	26.1 ± 3.3	26.0 ± 3.3	0.002
Ever-smoking	3,792 (59.9%)	5,096 (55.9%)	<0.001
Hypertension	3,999 (63.1%)	5,675 (62.2%)	0.242
Hyperlipidemia	5,243 (82.8%)	7,605 (83.4%)	0.336
Diabetes mellitus	2,176 (34.3%)	2,915 (31.9%)	0.002
Ischemic stroke	731 (11.5%)	996 (10.9%)	0.227
Renal insufficiency	113 (1.8%)	138 (1.5%)	0.189
Prior MI	1,631 (25.7%)	1,898 (20.8%)	<0.001
Prior PCI	1,192 (18.8%)	2,006 (22.0%)	<0.001
Prior CABG	205 (3.2%)	143 (1.6%)	<0.001
Prior gastrointestinal bleeding	227 (3.6%)	326 (3.6%)	0.973
Hemoglobin <11 g/dl	65 (1.0%)	97 (1.1%)	0.824
Platelet count <1,00,000/mm^3^	28 (0.4%)	39 (0.4%)	0.892
**Medication at discharge**			
Statin	5,415 (85.5%)	7,596 (83.3%)	<0.001
Beta-blocker	4,718 (74.5%)	6,436 (70.5%)	<0.001
ACEI/ARB	2,828 (44.6%)	3,763 (41.2%)	<0.001
**Angiographic and procedural characteristics**			
Multiple-vessel disease	3,638 (57.4%)	3,760 (41.2%)	<0.001
Diseased vessels per patient	2.5 ± 0.7	2.2 ± 0.8	<0.001
Lesions treated per patient	1.5 ± 0.7	1.2 ± 0.4	<0.001
Stents implanted per patient	2.4 ± 1.0	1.4 ± 0.5	<0.001
Total stent length per patient	64.9 ± 33.2	29.0 ± 12.6	<0.001
Bifurcation treated with 2 stents	1,065 (16.8%)	/	/
Left main as target vessel	762 (12.0%)	/	/
Bypass graft as target vessel	80 (1.3%)	/	/
Chronic total occlusion treated	2,159 (34.1%)	/	/

*PCI, percutaneous coronary intervention; MI, myocardial infarction; CABG, coronary artery bypass grafting; ACEI, angiotensin converting enzyme inhibitors; ARB, angiotensin II receptor blocker*.

Of all included patients, 13,383 patients (13.6%) received clopidogrel, and 2,076 (13.4%) received ticagrelor. A total of 579 (3.7%) patients switched between the two P2Y12 inhibitors during follow-up, of which 114 patients (5.5%) switched from ticagrelor to clopidogrel, and 465 patients (3.5%) switched from clopidogrel to ticagrelor. Ticagrelor was prescribed in 17.7% (*n* = 1,123) and 10.4% (*n* = 953) of patients with complex *vs*. non-complex PCI, respectively. Baseline characteristics for ticagrelor-treated and clopidogrel-treated patients stratified by procedural complexity are presented in Table 2. In both complex and non-complex PCI groups, ticagrelor-treated patients were younger and more likely to have hypertension but less likely to have a history of a previous ischemic stroke than clopidogrel-treated patients. Patients prescribed ticagrelor had a higher proportion of previous MI and PCI. In terms of procedural characteristics, ticagrelor-treated patients had a greater number of stents implanted and a greater total stent length compared with clopidogrel-treated patients.

**Table 2:** Baseline characteristics of patients treated with ticagrelor vs. clopidogrel in complex and non-complex PCI groups.

	**Complex PCI (*****n*** **=** **6,335)**			**Non-complex PCI (*****n*** **=** **9,124)**		
	**Ticagrelor (*n* = 1,123)**	**Clopidogrel (*n* = 5,212)**	**p Value**	**Ticagrelor (*n* = 953)**	**Clopidogrel (*n* = 8,171)**	***p* Value**
Age, years	57.4 ± 10.0	59.5 ± 10.1	<0.001	57.3 ± 9.8	60.0 ± 9.8	<0.001
Female	215 (19.1%)	1,034 (19.8%)	0.596	203 (21.3%)	2,011 (24.6%)	0.024
Body mass index, kg/m^2^	26.2 ± 3.2	26.1 ± 3.3	0.645	26.1 ± 3.3	26.0 ± 3.3	0.165
Ever-smoking	672 (59.8%)	3,120 (59.9%)	0.989	550 (57.7%)	4,546 (55.6%)	0.222
Hypertension	679 (60.5%)	3,320 (63.7%)	0.041	545 (57.2%)	5,130 (62.8%)	0.001
Hyperlipidemia	930 (82.8%)	4,313 (82.8%)	0.960	772 (81.0%)	6,833 (83.6%)	0.040
Diabetes mellitus	376 (33.5%)	1,800 (34.5%)	0.500	303 (31.8%)	2,612 (32.0%)	0.914
Ischemic stroke	87 (7.7%)	644 (12.4%)	<0.001	83 (8.7%)	913 (11.2%)	0.021
Renal insufficiency	18 (1.6%)	95 (1.8%)	0.614	14 (1.5%)	124 (1.5%)	0.908
Prior MI	364 (32.4%)	1,267 (24.3%)	<0.001	364 (38.2%)	1,534 (18.8%)	<0.001
Prior PCI	283 (25.2%)	909 (17.4%)	<0.001	304 (31.9%)	1,702 (20.8%)	<0.001
Prior CABG	29 (2.6%)	176 (3.4%)	0.172	18 (1.9%)	125 (1.5%)	0.398
Prior gastrointestinal bleeding	33 (2.9%)	194 (3.7%)	0.200	24 (2.5%)	302 (3.7%)	0.064
Hemoglobin <11 g/dl	12 (1.1%)	53 (1.0%)	0.876	10 (1.0%)	87 (1.1%)	0.965
Platelet count <1,00,000/mm^3^	2 (0.2%)	26 (0.5%)	0.142	3 (0.3%)	36 (0.4%)	0.573
**Medication at discharge**						
Statin	923 (82.2%)	4,492 (86.2%)	0.001	730 (76.6%)	6,866 (84.0%)	<0.001
Beta-blocker	821 (73.1%)	3,897 (74.8%)	0.247	628 (65.9%)	5,808 (71.1%)	0.001
ACEI/ARB	525 (46.7%)	2,303 (44.2%)	0.117	394 (41.3%)	3,369 (41.2%)	0.947
**Angiographic and procedural characteristics**						
Multiple-vessel disease	657 (58.5%)	2,981 (57.2%)	0.421	415 (43.5%)	3,345 (40.9%)	0.121
Diseased vessels per patient	2.5 ± 0.7	2.5 ± 0.7	0.96	2.3 ± 0.8	2.2 ± 0.8	0.062
Lesions treated per patient	1.6 ± 0.7	1.5 ± 0.7	0.132	1.2 ± 0.4	1.2 ± 0.3	0.072
Stents implanted per patient	2.6 ± 1.0	2.4 ± 0.9	<0.001	1.4 ± 0.5	1.3 ± 0.4	<0.001
Total stent length per patient	68.4 ± 35.0	64.2 ± 32.7	<0.001	30.6 ± 12.9	28.8 ± 12.5	<0.001
Bifurcation treated with 2 stents	192 (17.1%)	873 (16.7%)	0.778	/	/	/
Left main as target vessel	177 (15.8%)	585 (11.2%)	<0.001	/	/	/
Bypass graft as target vessel	10 (0.9%)	70 (1.3%)	0.218	/	/	/
Chronic total occlusion treated	396 (35.3%)	1,163 (33.8%)	0.357	/	/	/

*PCI, percutaneous coronary intervention; MI, myocardial infarction; CABG, coronary artery bypass grafting; ACEI, angiotensin converting enzyme inhibitors; ARB, angiotensin II receptor blocker*.

### Clinical Outcomes of Patients Undergoing Complex or Non-complex PCI at 12 Months

The incidence rates of 1-year clinical outcomes in patients with and without complex PCI are reported in Table 3. The median follow-up period was 369 days (IQR: 365–378 days).

**Table 3:** Clinical outcomes at 1 year of patients undergoing complex vs. non-complex PCI.

	**All patients (*n* = 15,459)**	**Complex PCI (*n* = 6,335)**	**Non-complex PCI (*n* = 9,124)**	**Adjusted HR (95% CI)**
**MACE**	979 (6.3%)	679 (10.7%)	300 (3.3%)	3.510 (3.058–4.030)
Cardiac death	185 (1.2%)	105 (1.7%)	80 (0.9%)	2.063 (1.530–2.782)
Myocardial infarction	174 (1.1%)	102 (1.6%)	72 (0.8%)	2.242 (1.655–3.037)
Target vessel revascularization	746 (4.8%)	546 (8.6%)	200 (2.2%)	4.238 (3.593–4.997)
Major bleeding	93 (0.6%)	43 (0.7%)	50 (0.5%)	1.388 (0.922–2.091)
Minor bleeding	178 (1.2%)	98 (1.5%)	80 (0.9%)	2.042 (1.505–2.769)

*MACE, major adverse cardiac events; PCI, percutaneous coronary intervention; HR, hazard ratio*.

The composite primary efficacy endpoint occurred in 679 (10.7%) patients in the complex PCI group and 300 (3.3%) in the non-complex PCI group. By multivariate Cox modeling, complex PCI was independently associated with increased risk of MACE (adjusted HR: 3.510; 95% CI: 3.058 to 4.030; *p* < 0.001). Patients who underwent complex PCI were at significantly higher risk of individual primary outcomes, including cardiac death (adjusted HR: 2.063; 95% CI: 1.530 to 2.782; *p* < 0.001), myocardial infarction (adjusted HR: 2.242; 95% CI: 1.655 to 3.037; *p* < 0.001), and target vessel revascularization (adjusted HR: 4.238; 95% CI: 3.593 to 4.997; *p* < 0.001). By including each component of complex PCI as a variable within the same multivariate models separately, ≥ three stents implanted (adjusted HR: 1.451; 95% CI: 1.256 to 1.676; *p* < 0.001), bifurcation PCI with ≥ two stents (adjusted HR: 1.419; 95% CI: 1.145 to 1.759; *p* = 0.001), total stent length > 60 mm (adjusted HR: 4.370; 95% CI: 3.850 to 4.961; *p* < 0.001), and chronic total occlusion as target lesions (adjusted HR: 2.588; 95% CI: 2.247 to 2.980; *p* < 0.001) were independent predictors of MACEs after procedure, while surgical bypass graft as target lesions (*p* = 0.154), unprotected left main PCI (*p* = 0.128), ≥ three lesions treated (*p* = 0.584), and three vessels treated (*p* = 0.629) showed no significant association with risk of MACEs.

There were no statistically significant differences in the risk of major bleeding events (*p* = 0.116) between patients with complex and non-complex PCI groups, whereas complex PCI caused an increased risk of minor bleeding events (adjusted HR: 2.042; 95% CI: 1.505–2.769; *p* < 0.001).

### Clinical Outcomes of Patients Treated With Ticagrelor or Clopidogrel at 12 Months

**Figure 2:**
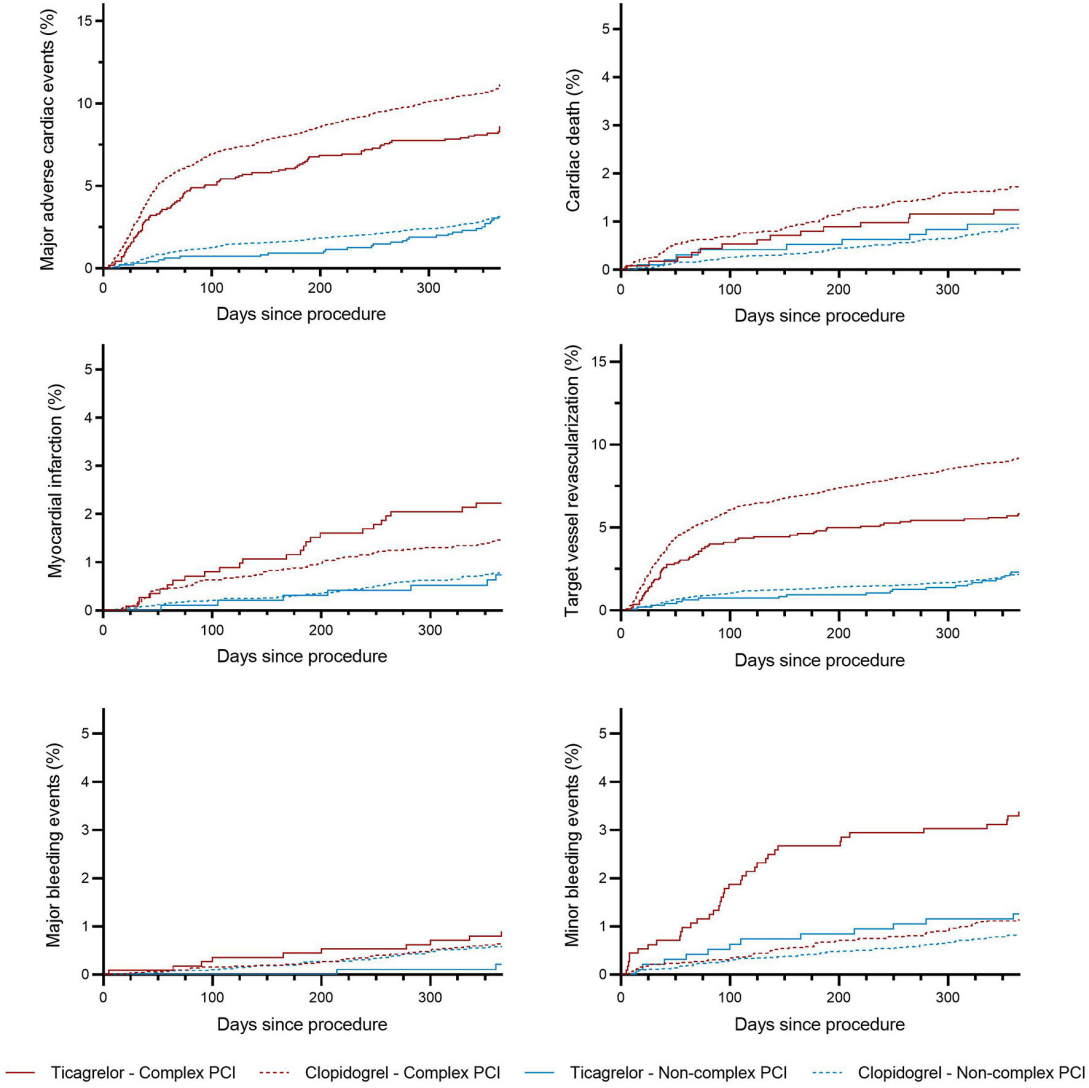
Incidence curves for the endpoints of patients stratified by P2Y12 inhibitors and complex PCI. Kaplan–Meier curves of patients stratified according to P2Y12 inhibitors and complex percutaneous coronary intervention for endpoints, including major adverse cardiac events, cardiac death, myocardial infarction, repeat target vessel revascularization major bleeding, and minor bleeding.

The incidence rates of 1-year clinical outcomes of patients treated with ticagrelor *vs*. clopidogrel in complex and non-complex PCI groups are summarized in Table 4. Kaplan–Meier curves for MACEs, individual primary efficacy endpoints, and bleeding outcomes are presented in Figure 2.

**Table 4:** Clinical outcomes at 1 year of patients treated with ticagrelor vs. clopidogrel in complex and non-complex PCI groups.

	**Complex PCI (*****n*** **=** **6,335)**			**Non-complex PCI (*****n*** **=** **9,124)**			
	**Ticagrelor** **(*n* = 1,123)**	**Clopidogrel** **(*n* = 5,212)**	**Adjusted HR (95% CI)**	**Ticagrelor** **(*n* = 953)**	**Clopidogrel** **(*n* = 8,171)**	**Adjusted HR (95% CI)**	***p* Value for interaction**
**MACE**	97 (8.6%)	582 (11.2%)	0.764 (0.615–0.949)	29 (3.0%)	271 (3.3%)	0.916 (0.621–1.351)	0.001
Cardiac death	14 (1.2%)	91 (1.7%)	0.654 (0.371–1.153)	9 (0.9%)	71 (0.9%)	0.955 (0.472–1.932)	0.989
Myocardial infarction	25 (2.2%)	77 (1.5%)	1.450 (0.919–2.288)	7 (0.7%)	65 (0.8%)	0.893 (0.405–1.968)	<0.001
Target vessel revascularization	66 (5.9%)	480 (9.2%)	0.631 (0.485–0.820)	22 (2.3%)	178 (2.2%)	1.122 (0.716–1.760)	0.047
Major bleeding	10 (0.9%)	33 (0.6%)	1.573 (0.767–3.228)	2 (0.2%)	48 (0.6%)	0.420 (0.101–1.740)	0.075
Minor bleeding	38 (3.4%)	60 (1.2%)	3.099 (2.049–4.687)	12 (1.3%)	68 (0.8%)	1.656 (0.887–3.095)	<0.001

*MACE, major adverse cardiac events; PCI, percutaneous coronary intervention; HR, hazard ratio*.

**Figure 3:**
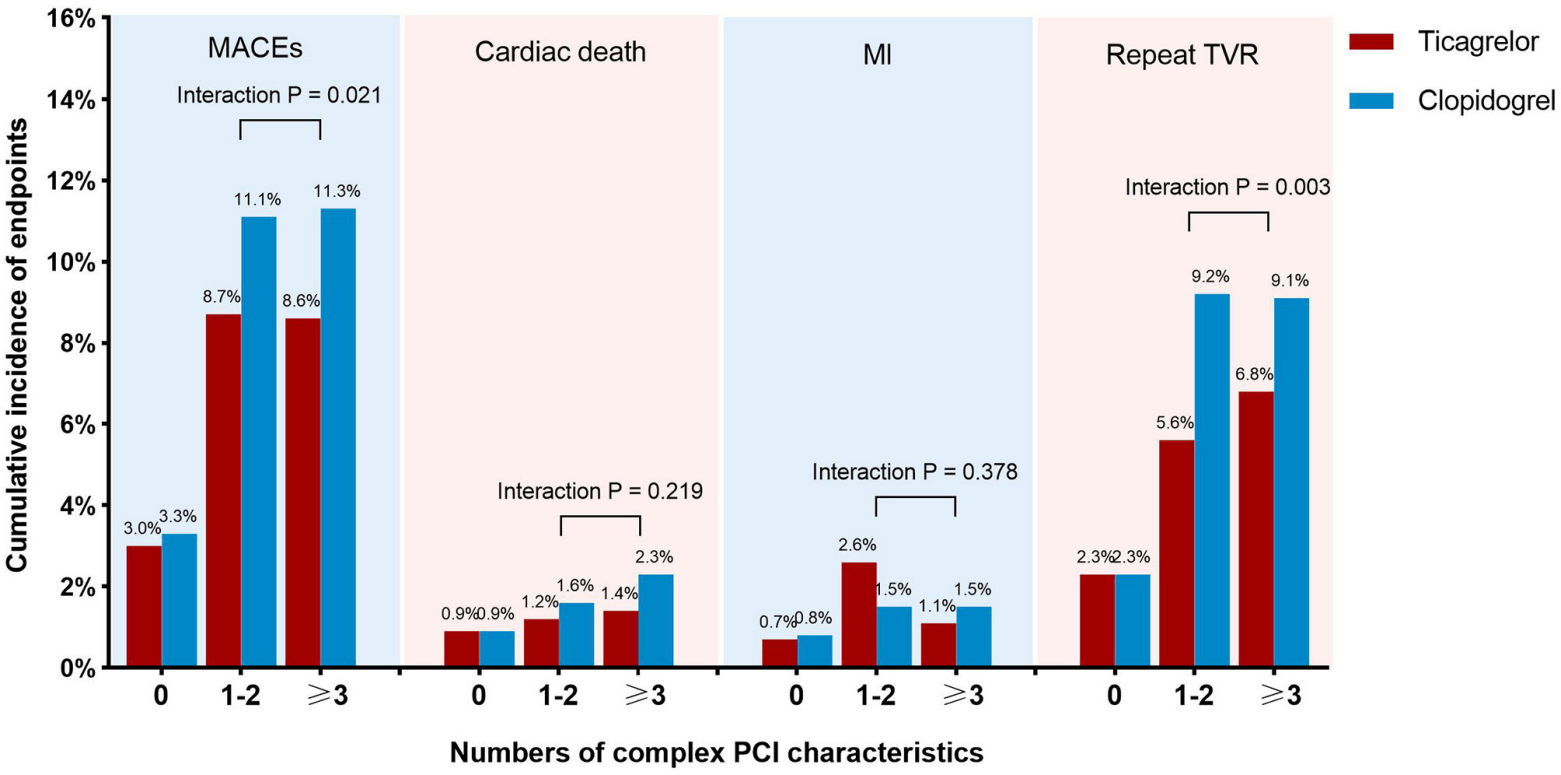
Incidence of clinical outcomes at 1 year of patients treated with ticagrelor *vs*. clopidogrel stratified by number of complex PCI characteristics. The incidences of endpoints, including major adverse cardiac events, cardiac death, myocardial infarction, and repeat target vessel revascularization, were compared between patients treated with ticagrelor and clopidogrel among subgroups of patients with 0, 1–2, or 3 or more complex PCI characteristics.

The primary efficacy endpoint occurred significantly less frequently in patients-prescribed ticagrelor than clopidogrel in the complex PCI group (8.6 vs. 11.2%). As compared with clopidogrel, treatment with ticagrelor was associated with a significant reduction in risk of the primary efficacy endpoint (adjusted HR: 0.764; 95% CI: 0.615 to 0.949; *p* = 0.015) with treatment effect in favor of patients undergoing complex PCI (*p* for interaction = 0.001). Similarly, ticagrelor resulted in lower rates of target vessel revascularization (adjusted HR: 0.631; 95% CI: 0.485 to.820; *p* = 0.001) among patients who received complex PCI but not among patients who received non-complex PCI (*p* for interaction = 0.047). The risk of cardiac death was numerically lower but not statistically significant (*p* = 0.069) in ticagrelor-treated patients from the complex PCI group. The risk of myocardial infarction did not differ significantly (*p* = 0.113) between the ticagrelor- and clopidogrel-treated patients after complex PCI, while a numerical increase in the risk of myocardial infarction was found in patients receiving ticagrelor after complex PCI, as shown in Table 4. The benefit of treatment with ticagrelor *vs*. clopidogrelon MACEs was progressively greater in patients with more complexity characteristics (*p* for interaction = 0.021; Figure 3).

Regarding safety outcomes, no significant difference in the rates of major bleeding mainly including gastrointestinal hemorrhage and intracranial hemorrhage was found between the two treatment groups in patients who underwent complex PCI (*p* = 0.211). However, there was a significantly higher frequency of minor bleeding in the ticagrelor group than in the clopidogrel group (adjusted HR: 3.099; 95% CI: 2.049 to 4.687; *p* < 0.001) among patients with complex PCI (*p* for interaction < 0.001).

The sensitivity analysis showed that the treatment effect of ticagrelor was consistent when patients (*n* = 579) switching between the two P2Y12 inhibitors during follow-up were excluded (adjusted HR: 0.743; 95% CI: 0.590 to 0.936; *p* = 0.012; *p* for interaction = 0.005).

## Discussion

The present study enrolled 15,459 patients with SCAD from a retrospective registry and assessed the efficacy and safety of ticagrelor *vs*. clopidogrel according to PCI complexity. The results suggested that the patients who underwent complex PCI had a significantly increased adjusted risk of ischemic events as compared with those who underwent non-complex PCI. Treatment with ticagrelor was more effective in preventing composite adverse outcomes, including cardiac death, MI, and repeat target vessel revascularization than clopidogrel among patients with complex PCI, but not among patients with non-complex PCI. Moreover, the beneficial effect of ticagrelor over clopidogrel was achieved without an increase in the risk of major bleeding but with a significant increase in the risk of minor bleeding.

The optimal antiplatelet therapy after PCI for stable CAD is still under debate, while ticagrelor has been proved to be superior to clopidogrel in patients with ACS (12, 13). Whether ticagrelor is also superior to clopidogrel for the prevention of ischemic events in patients presenting with SCAD remains uncertain. There are few dedicated randomized clinical trials investigating the benefit of ticagrelor or prasugrel over clopidogrel in patients with SCAD. The ALPHEUS study, which is the largest randomized prospective trial focusing on the DAPT in patients with stable coronary, found that ticagrelor showed no difference compared with clopidogrel in reducing periprocedural myocardial necrosis within 48 h of high-risk elective PCI (14). Nevertheless, results of the ALPHEUS study support the safety of ticagrelor in patients with SCAD by confirming the fact that treatment with ticagrelor did not cause an increase in major bleeding despite a higher level of platelet inhibition. The SASSICAIA trial investigated the impact of stronger platelet inhibition by an intensified oral loading strategy with prasugrel 60 mg *vs*. standard loading strategy with clopidogrel 600 mg among patients undergoing elective PCI. The results suggested that prasugrel-based and clopidogrel-based loading strategies had similar efficacy and safety (15). A subgroup analysis of the GLOBAL LEADERS trial found that there was no difference on treatment effects between patients with ACS and SCAD treated with ticagrelor monotherapy, following 1-month DATP, but revealed a non-significant increase in the risk of bleeding with the ticagrelor monotherapy in patients with SCAD compared with standard DAPT (16).

Data on long-term benefit of ticagrelor after elective PCI among patients with SCAD are quite scarce. Our previously published research, a real-world observational study comparing the treatment effect of ticagrelor and clopidogrel in patients with SCAD treated with PCI, suggested that the use of ticagrelor instead of clopidogrel was associated with a lower rate of MACEs within 1 year after PCI by performing propensity score matching in a retrospective cohort of 9,379 consecutive patients with SCAD (17). We also observed that the benefits of ticagrelor in significantly reducing the risk of ischemic events after PCI were consistent between patients with SCAD and patients with ACS among carriers of two CYP2C19 loss-of-function alleles who were common in east Asians (18).

Based on some subgroup analyses from pertinent randomized clinical trials, clopidogrel in addition to aspirin is generally recommended in patients with SCAD for 6 months, following coronary stenting in current guidelines (3). Remarkably, the practice guidelines state that ticagrelor or prasugrel on top of aspirin instead of clopidogrel may be considered in specific high-risk situations of elective stenting, including complex PCI procedures, such as left main stenting and chronic total occlusion procedures in patients with SCAD (1, 3). Our study showed a significant benefit of stronger P2Y12 inhibition using ticagrelor compared with clopidogrel to prevent adverse events in patients with SCAD with complex PCI, which is aligned with the recommendations of current guidelines. Our results also suggested that patients treated with ticagrelor had higher proportions of previous MI and revascularization, more stents implanted, and longer total stent length compared with patients treated with clopidogrel. Hence, increased procedural complexity, more extensive CAD, and high burden of comorbidities were properly considered to be reasons for ticagrelor use in patients with SCAD undergoing complex PCI.

Despite significant reduction in rates of PCI procedures, the rate of complex PCI has continued to increase over the past decades. In previous studies, the increment of procedural complexity has been shown to be associated with increased ischemic risk, particularly in the first year after PCI (19). Moreover, the PCI complexity has been proposed to be an important parameter to take into account for clinical decision-making on DAPT regimens. A *post-hoc* patient-level pooled analysis of six randomized control trials suggested that, compared with a short period of DAPT, 1 year or more of DAPT after PCI could substantially reduce the risk of ischemic events with a magnitude that was greater in patients with more complex angiographic features (7). In addition, a study exploring the relationship between PCI complexity and occurrence of adverse events showed that the impact of complex PCI was consistent in patients with SCAD *vs*. ACS and even greater in patients with SCAD (20). Hence, it is reasonable to assume that patients with SCAD undergoing complex PCI may benefit by more potent antiplatelet therapy.

Patients who undergo complex PCI are reported to have more advanced CAD and higher burden of comorbidities, which are related to both ischemic and bleeding risks (9, 21, 22). It is noticeable that identifying the optimal antiplatelet therapy for patients undergoing complex PCI remains challenging. Our study provided information for efficacy and safety of intensified DAPT in patients with SCAD with high ischemic risk features. In the present analysis, we observed that ticagrelor decreased the risk of MACEs in patients undergoing complex PCI, which was driven by a significant reduction in risk of target vessel revascularization compared with clopidogrel. The incidence of myocardial infarction in our study tended to be numerically higher in ticagrelor-treated patients, which might be partly explained by the great procedural complexity of patients receiving ticagrelor than clopidogrel. In addition to intensified DAPT, patients undergoing complex PCI who did not fulfill the criteria of high bleeding risk could significantly benefit from prolonged DAPT, while patients with high bleeding risk could not (23).

With regard to the bleeding risk after procedure, some studies detected a strong relationship between complex PCI and an increased risk of bleeding events (20, 24), while some studies reported that PCI complexity might not be associated with a higher bleeding risk (7, 19). It was reported that the increased risk of ischemic events and mortality in patients who underwent complex PCI were further increased in those with >one element of the complexity criteria. However, the fulfillment of complex PCI criteria did not significantly affect bleeding risk (23). The fact that the correlation between PCI complexity and bleeding risk remains controversial might be explained in part by various definitions of complex PCI and different study populations across different studies (24). Although ticagrelor was considered to be associated with higher bleeding risk, the DAPT type (clopidogrel vs. ticagrelor) had no significant impact on risk of bleeding events at 1 year after PCI in patients with high bleeding risk (25).

The increased risk of bleeding remains an important concern for patients treated with DAPT after stent implantation (26), especially in east Asian patients who have a higher risk of severe bleeding risk than the western population (27, 28). In several previous studies enrolling east Asians, the benefits of stronger P2Y12 inhibitors were partly counterbalanced by an increased risk of bleeding events, which was related to a higher risk of mortality (29–31). The recommendations for use of ticagrelor over clopidogrel in patients with ACS are based on the clinical trials from western countries and need to be further validated in east Asians. An individual assessment of bleeding and ischemic risk and a careful evaluation of the clinical and anatomic profile of a patient seem to be necessary when tailoring DAPT. Numerous risk stratification tools have been proposed to help identify patients at high risk of bleeding and ischemic events (26, 32). The guidelines have emphasized the need to individualize the DAPT intensity and duration on the basis of ischemic and bleeding risk factors in order to improve clinical outcomes (1, 3, 33). The findings of our study supported the superiority of ticagrelor *vs*. clopidogrel in anti-ischemic efficacy after complex PCI, using data from an East Asian cohort. Meanwhile, regarding bleeding risk, we found that ticagrelor did increase the rate of minor bleeding events but not the rate of major bleeding events.

In recent years, P2Y12 inhibitor monotherapy following DAPT after PCI has attracted a lot of attention, which was shown to be associated with a lower risk of major bleeding and net adverse clinical events compared with traditional DAPT (9, 34). A meta-analysis of randomized trials investigating P2Y12 inhibitor monotherapy after revascularization suggested that the benefit of P2Y12 inhibitor monotherapy on lower bleeding rates was consistent in subgroup analyses according to complexity of PCI (complex vs. non-complex PCI) and clinical presentation (stable CAD vs. ACS) (35).

Besides event rates, the costs and quality of life should also be taken into consideration when selecting P2Y12 inhibitors for patients treated with PCI, especially when ticagrelor was more expensive than standard clopidogrel in most regions. The cost-effectiveness of different treatment strategies should be assessed and compared so as to prioritize treatments among limited health-care resources. Although ticagrelor has been proved to be a cost-effective treatment for patients with ACS compared to clopidogrel in several health economic evaluations (36–38), its long-term cost-effectiveness among patients with SCAD is still uncertain, and a dedicated study on cost-effectiveness analysis is needed to help optimize the decision-making process of DAPT for patients with SCAD.

The present study has some limitations. First, this was an observational retrospective study from a registry with its inherent limitations such as the non-randomized treatment selection of P2Y12 inhibitors. Despite use of careful multivariate adjustments to lessen the potential confounding effects related to differences across groups, residual confounders cannot be entirely excluded. Second, the generalizability of our findings is limited due to the single-center design of our study. However, our center is the National Center for Cardiovascular Disease of China, and the patients included in this study come from all over the country, which could cover a representative sample of the study population. Third, there is no universal definition for complex PCI. We used a widely accepted definition of complex PCI, which has been described in previous studies (7, 9). Finally, the follow-up period of our study was short, and enrolled patients were followed up for only 1 year. However, a previous study has demonstrated that the association between complex coronary artery lesions and higher risk of subsequent adverse events was substantially attenuated after the 1st year of procedure (19). Furthermore, DAPT is generally recommended for 6 to 12 months in patients with SCAD treated with PCI. Therefore, a 1-year follow-up period is reasonable for the present study, which compared different DAPT regimens in patients with SCAD undergoing PCI.

In conclusion, patients who undergo complex PCI are at an increased risk of adverse ischemic events. Among patients with stable coronary artery disease undergoing complex PCI, treatment with ticagrelor is associated with a lower risk of MACEs within 12 months after procedure but did not cause an increase of major bleeding events *vs*. clopidogrel. Our findings provide information for tailoring DAPT and support the use of ticagrelor in patients with stable coronary artery disease. Further research on optimal antiplatelet therapy in this population is needed to determine whether they can benefit from potent P2Y12 inhibitors as compared with routine use of clopidogrel.

## Data Availability Statement

The raw data supporting the conclusions of this article will be made available by the authors, without undue reservation.

## Ethics Statement

The studies involving human participants were reviewed and approved by the Ethics Committee of Fuwai Hospital. Written informed consent for participation was not required for this study in accordance with the national legislation and the institutional requirements.

## Author Contributions

ZX, JL, and HQ contributed to conception and design of the study. TG and YWa organized the database. ZX performed the statistical analysis and wrote the first draft of the manuscript. JZ and YL wrote sections of the manuscript. All authors contributed to manuscript revision, read, and approved the submitted version.

## Conflict of Interest

The authors declare that the research was conducted in the absence of any commercial or financial relationships that could be construed as a potential conflict of interest.

## Publisher's Note

All claims expressed in this article are solely those of the authors and do not necessarily represent those of their affiliated organizations, or those of the publisher, the editors and the reviewers. Any product that may be evaluated in this article, or claim that may be made by its manufacturer, is not guaranteed or endorsed by the publisher.
